# Biochemical Markers of Physical Exercise on Mild Cognitive Impairment and Dementia: Systematic Review and Perspectives

**DOI:** 10.3389/fneur.2015.00187

**Published:** 2015-08-26

**Authors:** Camilla Steen Jensen, Steen Gregers Hasselbalch, Gunhild Waldemar, Anja Hviid Simonsen

**Affiliations:** ^1^Department of Neurology, Danish Dementia Research Centre, Rigshospitalet – Copenhagen University Hospital, Copenhagen, Denmark; ^2^Department of Clinical Medicine, University of Copenhagen, Copenhagen, Denmark

**Keywords:** dementia, MCI, exercise intervention, biomarkers, physical activity

## Abstract

**Background:**

The cognitive effects of physical exercise in patients with dementia disorders or mild cognitive impairment have been examined in various studies; however the biochemical effects of exercise from intervention studies are largely unknown. The objective of this systematic review is to investigate the published results on biomarkers in physical exercise intervention studies in patients with MCI or dementia.

**Methods:**

The PubMed database was searched for studies from 1976 to February 2015. We included intervention studies investigating the effect of physical exercise activity on biomarkers in patients with MCI or dementia.

**Results:**

A total of eight studies were identified (*n* = 447 patients) evaluating exercise regimes with variable duration (single session–three sessions/week for 26 weeks) and intensity (light-resistance training–high-intensity aerobic exercise). Various biomarkers were measured before and after intervention. Seven of the eight studies found a significant effect on their selected biomarkers with a positive effect of exercise on brain-derived neurotrophic factor, cholesterol, testosterone, estradiol, dehydroepiadrosterone, and insulin in the intervention groups compared with controls.

**Conclusion:**

Although few studies suggest a beneficial effect on selected biomarkers, we need more knowledge of the biochemical effect of physical exercise in dementia or MCI.

## Introduction

The prevalence of dementia is increasing, currently affecting more than 44 million people, and estimated affect 75 million people worldwide by 2030. Alzheimer’s disease (AD) accounts for the majority of dementia cases (1–3). Currently there is no cure for these disorders, and there are currently no effective pharmacological interventions (4, 5). Attention has therefore turned toward non-pharmacological approaches, including exercise, to slow the cognitive decline associated with dementia (2, 6). Linking evidence from population-based cohorts or RCT studies with biochemical evidence will be crucial in order to understand how non-pharmacological interventions may potentially alter the course of the disease.

In epidemiological studies, retrospective cohort studies, and case–control studies, there is consensus that an active lifestyle in midlife decreases the risk of dementia in late adulthood (7, 8). The cognitive effects of physical exercise and an active lifestyle in healthy elderly subjects, and in those with MCI and dementia, have also been examined in various cross-sectional studies, intervention studies, and prospective studies, with conflicting results (9–21). Almost all studies in patients with mild cognitive impairment (MCI) show some effect on cognition, but recent systematic reviews call for caution when interpreting results in dementia due to limited evidence (22, 23). Lack of consensus could be due to differences in the study methodologies used, type of physical activity, or in the cognitive measures used.

Because some studies have identified a clinical effect of physical exercise, it is imperative to understand if and how exercise alters the pathophysiology of dementia. Such an understanding is necessary for the successful promotion and implementation of physical exercise as a part of the treatment for dementia. Our current knowledge comes largely from animal studies. Beta-Amyloid (Aβ) pathology can be altered in response to exercise in a mouse and rat model for AD (24, 25), and brain plasticity proteins, like brain-derived neurotrophic factor (BDNF), can be up-regulated in response to physical exercise (26). Also, long-term exercise treatment reduces oxidative stress (OX) in the hippocampus of aging rats (27). In a large study of healthy elderly subjects, lower plasma and brain Aβ was observed in those reporting higher levels of physical activity (21), and similar findings has been found in preclinical AD subjects (28), consistent with animal studies suggesting that physical activity may modulate specific AD pathology in humans as well. However, because observational and cross-sectional designs cannot establish causality, we need randomized controlled intervention trials to understand the biochemical effects of exercise.

Exercise-based interventions studies in various diseases have clarified some of the biochemical effects of physical activity, such as improved metabolic homeostasis in diabetes mellitus (29), reduced OX in obese subjects (30), and reduced low-grade inflammation in coronary artery disease (31). Thus, physical exercise may exert its effect through modulation of specific AD pathology and/or through pathological processes common to other diseases.

Therefore, the object of this study was to systematically review and evaluate the scientific literature regarding the biochemical effect of exercise in MCI and dementia disorders in intervention trials and furthermore to provide recommendations for future biochemical studies in this field. Based on the studies cited above, we hypothesized that physical exercise interventions would improve not only specific Aβ pathology, but also pathological processes downstream of Aβ accumulation.

## Methods

This systematic review was performed according to the recommendation of the Cochrane collaboration (32) and the Preferred Reporting Items for Systematic Review and Meta-Analysis: the PRISMA statement (33).

### Eligibility criteria

Randomized controlled trials or clinical trials investigating the effect of physical exercise or activity on patients with MCI or dementia were selected to review. Studies must have obtained bio-fluid markers, regardless of whether the biomarkers were included as primary or secondary outcome.

### Search strategy

The following electronic database was searched: MEDLINE (accessed via PubMed). The database PubMed was selected because it contains more that 23 million citations from biomedical literature from MEDLINE, life science journals and online books.

The search conducted in February 2015 searched databases for the following MeSH terms and their English synonyms. Studies published from 1976 to 2015 were included.

Medline (Via Pubmed.org) was searched with the keywords and Boolean operators with the filter English and Human:

(“Dementia”[Majr]) AND (“Exercise”[Majr])
(“Mild Cognitive impairment”[Majr]) AND (“Exercise”[Majr])
(“Dementia”[Majr]) AND (“physical fitness”[Majr])
(“Mild Cognitive impairment”[Majr]) AND (“Physical fitness”[Majr])

**Figure 1:**
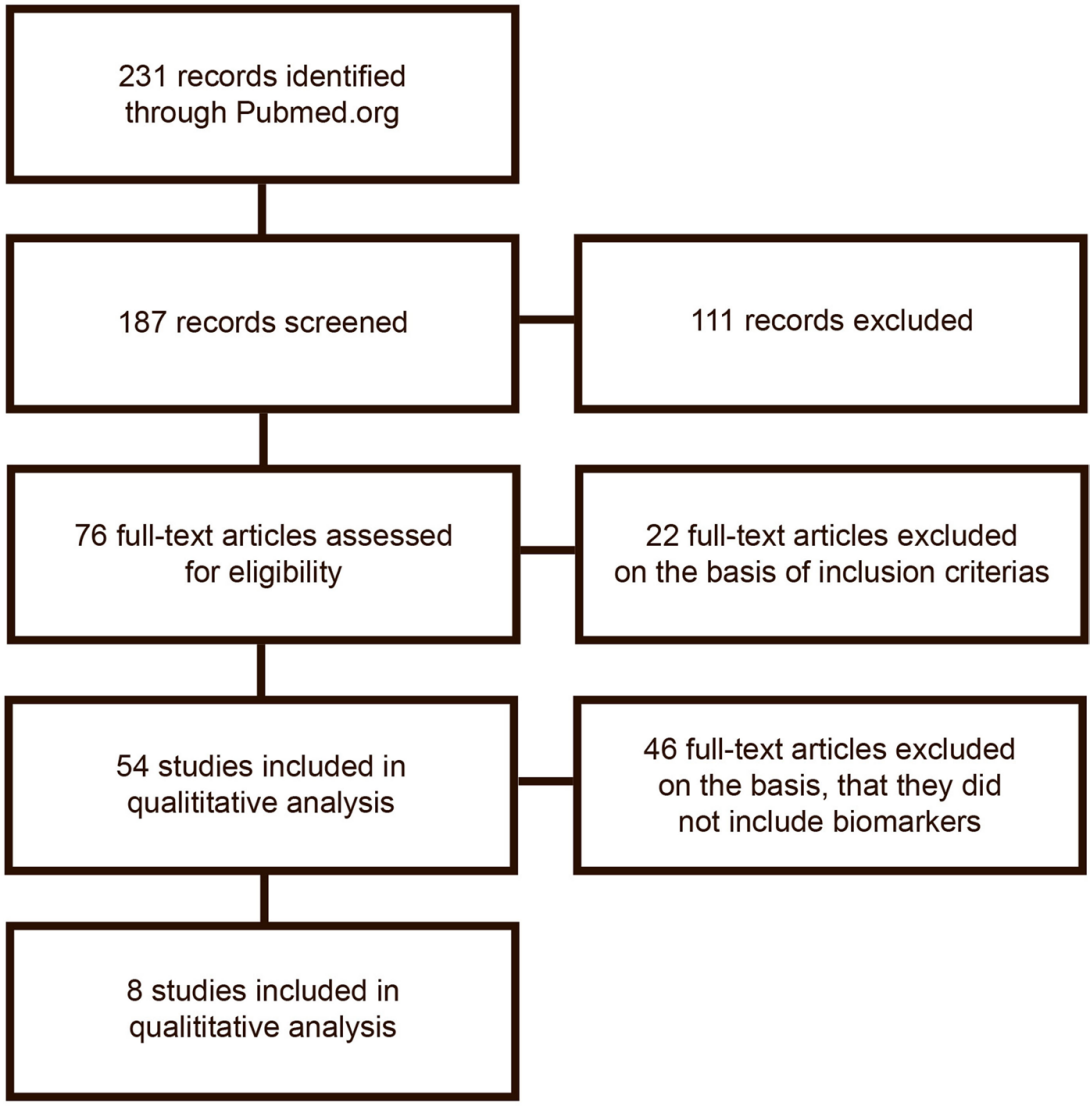
**Flowchart of the publication search and selection process**.

The search was done by two authors separately (first and second) author. The search results are described in Figure 1.

Inclusion criteria included: original work (no review or meta-analysis), physical activity/exercise as intervention, only full-text publication, and English language.

### Study selection and data extraction

Studies were selected on the basis of the inclusion criteria listed above. The selected studies are listed in Table 1 and Table S1 in Supplementary Material. Data extraction was done by the first author according to the data extraction form seen in Table S2 in Supplementary Material, in regards to author, endpoints measured, subjects, intervention, and results found.

**Table 1:** **Studies chosen for review**.

Publication	Endpoint	Subjects	Intervention	Results on cognition	Results biomarkers
Akishita et al. (15)	Cognitive markers ADL MMSE Blood markers: Estradiol Testosterone DHEA DHEA sulfate Sex hormone-binding globulin	13 ♀ AD^a^ Nursing home residents Mean age: 84.5 ± 5 Mean MMSE: 13.9 ± 1.9	*N* = 13, resistance training (light) + stretching 30 min 2/week^c^ + 5/week^b^ × 12 weeks	No change in MMSE No change in ADL	↑ Testosterone ↑ Estradiol ↑ DHEA
Baker et al. (34)	Cognitive markers: Symbol-digit modalities Verbal fluency Trail B STROOP Task switching Story recall List-learning Blood markers: Insulin IGF-1 Cortisol BDNF Platelet factor 4 Aβ_42_ Cholesterol Cardio markers: VO_2_ peak	29 MCI^a^ Home living Mean age Stretching 66 ± 6.0 Intervention 71 ± 7.5 Mean MMSE Stretching 28.8 ± 1.0 Intervention 28.6 ± 1.2	*n* = 10, stretching (<50% HRR) 45–60 min 4/week × 26 weeks^c^ *n* = 19, aerobic exercise (75–85% HRR) 45–60 min 4/week × 26 weeks^c^	♀ Increased executive function Increased performance in STROOP Both Increased performance in trail B	Gender difference ♀ ↑ Insulin sensitivity ↑ Insulin ↑ Cortisol in control group ↑ BDNF ♂ ↓ Cortisol in controls group ↓ BDNF ↑ IGF-1 Both ↓ Body fat ↓ Total Cholesterol ↑ VO_2_ peak
Cheng et al. (35)	Cognitive markers: MMSE Call-recall Categorical fluency Digit span Blood markers: Cholesterol HDL Tri-glycerides Glucose Genetic markers: ApoE genotype	110 dementia^a^ Nursing home residents Mean age Control 80.8 ± 7.2 Mahjong 81.9 ± 6.2 Tai Chi 81.8 ± 7.4 Mean MMSE Control 18.8 ± 4.1 Mahjong 19.0 ± 3.2 Tai Chi 18.7 ± 3.9	*n* = 39, aerobic exercise (Tai Chi) 60 min 3/week × 12 weeks^b^ *n* = 36, cognitive activity (Mahjong) 60 min 3/week × 12 weeks^b^	Tai Chi Slower decline in cognitive measures Mahjong Slower decline in cognitive measures	Non-reported
Coelho et al. (36)	Blood markers: BDNF	21 AD^a^ 18 cognitive normal (used as controls) Home living Mean age AD 76.3 ± 6.2 Controls 74.5 ± 4.7 Mean MMSE AD 21.0 ± 3.9 Controls 28.0 ± 2.5	*n* = 39, acute aerobic exercise (85% HRR) 17–22 min once^c^	None studied	Both: ↑ BDNF
Eggermont et al. (37)	Cognitive markers MMSE Face recognition Picture recognition Delayed word call Digit span backwards Category fluency Letter fluency Genetic markers ApoE	97 dementia^a^ Nursing home residents Mean age: 85.4 Mean MMSE: 17.7	*n* = 51, walking 30 min 5/week × 6 weeks^b^	No significant results reported	No significant results reported
Mancuso et al. (38)	Blood markers: COX Lactate	18 AD^a^ 20 cognitive normal No mention of living status Mean age AD 66.4 ± 8.9 Controls 63.4 ± 9.1 Mean MMSE AD 17.4 ± 4.9 Controls, not reported	*n* = 38, acute resistance training (increment 10–70% 1RM) once^b^	Not studied	↑ Lactate ↓ Lactate recovery after exercise ↓ COX activity
Nascimento et al. (39, 41)	Cognitive markers: MoCA Blood markers BDNF TNF-α IL-6	37 MCI^a^ 30 cognitive normal Home living Mean age MCI used as controls 68.5 ± 5.9 MCI exercise 67.3 ± 5.3 Cognitive normal used as controls 68.1 ± 5.7 Cognitive normal exercise 66.6 ± 7.9 Median MoCA MCI used as controls 21 MCI exercise 20 Cognitive normal used as controls 27 Cognitive normal exercise 27	*n* = 35 (20 MCI, 15), aerobic exercise (60–80% HRR) 60 min 3/week × 16 weeks^c^	MCI: increased cognition	Both ↓ TNF ↓ IL
Segal et al. (41)	Cognitive marker: IAPS Saliva markers: alpha-amylase	23 aMCI^a^ 31 cognitive normal No mentioning of home status Mean age aMCI 71.4 ± 2.4 Cognitive normal 69 ± 2 Mean MMSE aMCI, not reported Cognitive normal, not reported	*n* = 26 (11a MCI, 15 controls) acute aerobic exercise (70% VO_2max_) 6 min once^b^	Both: increased picture recall	Both: ↑ saliva alpha-amylase

*ADL, activities of daily living; DHEA, dehydroepiadrosterone; AD, Alzheimer’s disease; aMCI, amnestic mild cognitive impairment; MCI, mild cognitive impairment; IGF-1, insulin-like growth factor 1; BDNF, brain-derived neurotrophic factor; Aβ_40+42_, beta-amyloid isoform 40 and 42; VO_2_ peak, peak oxygen uptake; HRR, heart rate reserve; ♀, women; ♂, men; MMSE, mini-mental state examination; HDL, high-density lipoprotein; COX, cytochrome c oxidase; MoCA, Montreal Cognitive Assessment; TNF-a, tumor necrosis factor alpha; IL-6, interleukin-6; IPAS; International Affective Picture Set*.

*^a^Diagnosed according to international guidelines*.

*^b^Supervised by caregiver, healthcare worker, or not mentioned*.

*^c^Supervised by trainer or physiotherapist*.

## Results

The initial search gave 228 publications, from which 187 were collected for further reading and 111 of which were excluded due to irrelevance or because they did not meet the inclusion criteria on the basis of their title or abstract. From the remaining 76 publications, 54 met inclusion and exclusion criteria and were selected for analysis. After a detailed analysis, publications were excluded if they did not include analysis of biomarkers. The excluded publications are listed in Table S1 in Supplementary Material. In total, eight publications remained for inclusion in the review. Publications included are listed in Table 1. Figure 1 shows the flowchart of the data gathering process.

### Sample subjects

Although our MeSH term search covered all dementia diagnoses, the majority of identified publications studied patients with AD. Subjects were either from a nursing home-residing population (15, 35, 37) or a home-living population (34, 41, 42). Two studies did not describe living status (38, 41).

In Table 1, mean age and mean MMSE have been listed, giving a general indication of the sample subjects studied. The age range was from 66.4 to 85.4 years. The MMSE range was from 13.9 to 28.7. Only Baker et al. have a population with a MMSE above 21, which indicated that the majority of the studies have been on patients with moderate-to-severe dementia.

### Sample size

The numbers of subjects used in the selected studies range from 13 to 110 subjects. In general, the small sample sizes have generated little power for calculation of effect.

### Exercise protocol

Four studies implemented an aerobic training program with low-to-high intensity (34, 35, 37, 39), three studies investigated the effect of a single bout of high-intensity aerobic or resistance training exercise (38, 41, 42) and one study investigated the effect of light resistance training and stretching (15). Thus, most studies investigated aerobic training to investigate the effect on biochemical biomarkers. The studies reviewed applied very different training regimes, with regards to intensity, duration and frequency. Eggermont et al. (37) applied the lowest intensity with a walking program at a self-selected speed, and they did not report any significant results on any of their selected biomarkers. Cheng et al. applied a light exercise program and found that the exercise groups had a slower decline in their cognitive measures compared to controls. The three remaining aerobic exercise studies have applied a moderate-to-high-intensity exercise program, and they found a significant increase in levels of their selected biomarkers, and in the cognitive measures.

### Exercise supervision

Five of the eight selected studies had non-supervised training or supervision by caregivers. Three studies had supervision by trainers. Two studies reported use of heart rate monitors to ensure that the intended exercise intensity of the exercise was reached. In Baker et al. (34), only some of the training sessions were supervised.

### Cognition

Six out of these eight studies also investigated cognitive performance (15, 34, 35, 37, 39, 41). Of these six studies, four reported any significant effect on the cognitive measures. Baker et al. (34) reported an improvement in several tests of executive function, but only in women, Cheng et al. (35) reported a reduced decline in MMSE, Nascimento et al. (39) found an improved cognition measured by the Montreal Cognitive Assessment (MoCA), and Segal et al. (41) found a significant improved picture recall after exercise.

### Effect on biomarkers

In total, eight studies that focused on biochemical markers were identified. Seven out of eight studies investigated protein biomarkers (15, 34, 35, 38–42), and two studies investigated the difference in the effect of exercise depending on the patients ApoE genotype (35, 37). One study also investigated markers of cardiovascular health (34). Table 1 summarized the biochemical and cognitive findings.

In seven out of eight studies, a positive relationship was found between their selected biomarkers and the exercise intervention. Only one study did not find any significant results on any of their selected biomarkers (37).

Coelho et al. (36) and Segal et al. (41), found that exercise resulted in a significant up-regulation in the neuroplasticity protein BDNF. Baker et al. (34) also measured BDNF and found higher levels after exercise, however only in women. Nascimento et al. reported BDNF as one of their end points, however they did not report any findings. They did, however, report decreased Tumor necrosis factor alpha (TNF-α) and interleukin-6 (IL-6) levels after exercise.

Besides blood levels of BDNF, other biochemical compounds in blood including cholesterol and insulin were analyzed. Baker et al. (34) and Cheng et al. (35), measured plasma levels of insulin and cholesterol. Cheng et al. (35) also measured HDL, tri-glycerides, and glucose. Only Baker et al. (34) reported an effect of the intervention on these biomarkers, namely a significant decrease in cholesterol and an increase in insulin sensitivity.

On a different note besides neurological markers and metabolic markers, Akishita et al. (15) found that exercise had a significant up-regulating effect on selected sex hormones (testosterone, estradiol, and DEHA) in women. Segal et al. (41) found an increase in salivary alpha-amylase (sAA), an indirect measure of endogenous norepinephrine (NE), after a single session of high-intensity exercise (70% HRR). Mancuso et al. (38) found that lactate increased after exercise, and that platelet mitochondria COX activity was decreased.

We did not find any intervention studies that investigated the effect of exercise on established diagnostic markers for dementia disease, such as Aβ, tau, p-tau, and α-synuclein.

## Discussion

Physical exercise as a non-pharmacological treatment for medical disease has proven beneficial for reducing the risk for many diseases including stroke, high blood pressure, and mental disorders like chronic stress and depression (43). However, compared to our understanding or physical exercise’s impact on cardiovascular health and general fitness, our understanding of physical exercise’s impact on cognitive health is still very much in its infancy. An impact of physical exercise on quality of life and activity of daily living in patients with dementia has been established; however evidence of the molecular effects is not clear.

The objective of this study was to conduct a systematic review to identify and evaluate the scientific literature published on the effect of an exercise intervention in dementia, in regards to relevant biomarkers.

### Biochemical evidence of the effects of exercise

Insulin and diabetes have been connected to an increased risk of developing AD and cognitive impairment (44, 45). Two of the reviewed studies measured insulin sensitivity or glucose control. Baker et al. (34) found increased insulin sensitivity and increased insulin in the exercise group, however only in women. Cheng et al. (35) measured blood glucose levels, but they did not find any significant result.

Mancuso et al. (38) investigated the reactive oxygen species (ROS) generation and OX, measured via COX activity and lactate production in platelets. ROS and OX are thought to be involved in AD through the neurotoxicity of amyloid build up, metabolic impairments and free-radical production in mitochondria (46, 47). To investigate the metabolic contribution of mitochondrial impairment, COX activity and lactate production was measured before and after an exercise intervention. At baseline, the AD groups displayed higher levels of lactate and significantly lower activity of COX, compared to aged match cognitive normal individuals. This increased level of lactate in AD patients was unchanged throughout the exercise intervention, indicating mitochondrial impairment in AD. The exercise intervention did not alter COX activity, indicating that exercise might not be able to influence the mitochondrial electron transport chain (ETC). However, since Mancuso et al. (38) did not find any correlation with cognitive elements, such as MMSE, mitochondrial impairment might be an angle to study the pathology of AD, and not so much a way to improve cognitive decline.

Nascimento et al. (39, 41) investigated the influence of exercise on inflammation markers in MCI and cognitively normal subjects and found decreased levels of the pro-inflammatory cytokines, TNF-α and IL-6. Inflammation is a known factor in neurodegenerative diseases (48–50). Both pro-inflammatory cytokines (e.g., IL-6) and anti-inflammatory cytokines (e.g., IL-10 and IL-18) have been found to be increased in AD (51), and wherein it is speculated that an increased inflammatory response negatively contributes to neurodegeneration in AD (50). Several studies have shown that inflammation is directly influenced by physical activity, which down-regulates pro-inflammatory reactions in the brain (52). For further insight into the pathology of neuroinflammation, it might be beneficial to measure a variety of factors, both pro-inflammatory and anti-inflammatory.

Three of eight identified studies have focused on BDNF, all of which found an increase in BDNF after exercise. Lower levels of brain tissue BDNF have been seen in patients with AD compared to healthy controls (26, 53). The exercise-induced BDNF increase seen in the studies in this review has also been reported in animal studies, where brain levels of BDNF were increased after exercise (26), and in an intervention study in young healthy men, where plasma BDNF was increased with exercise. In order to achieve a more precise measurement of neuronal BDNF without the systemic component, BDNF levels in CSF could be assessed.

Alongside BDNF, Akishita et al. (15) measured increased levels of female sex hormones after exercise. Lower levels of sex hormones have previously been shown to increase the risk of AD (54). Exercise has been found to increase sex hormones and sex hormone-binding globulin in post-menstrual women (55–57). One could therefore speculate that an increase in sex hormones is beneficial to the cognitive performance in patients already diagnosed with AD. In the study by Akishita et al. (15), there was no effect on ADL or cognition, and the up-regulating effect of exercise on sex hormones was lost after 3 months post-exercise.

Pharmacological evidence established that NE is involved in memory modulation, and can be regulated by exercise (58–60). This makes NE modulation by exercise an ideal target for memory modulation in patients with cognitive impairments. Segal et al. (41) studied this relationship with a single bout of high-intensity exercise in patients with aMCI. The cognitive performance was investigated with picture recall before and after exercise, and NE was measured indirectly via sAA. They found that performance in picture recall was significantly improved in the exercise cognitive normal control group as well in the exercise aMCI groups, and not in the corresponding non-exercise groups. Furthermore, sAA levels were equally increased in both exercise groups (cognitive normal and aMCI). When it comes to dementia diseases, like AD with more advanced neurodegeneration, it is unknown if exercise is able to up-regulate NE, so further studies are needed. In addition, the potential harms of recurring acute increases in NE need to be investigated.

None of the review studies in this review focused their attention on already previously established markers of neurodegenerative disease. Baker et al. (61) has studied the effect of a diet intervention with or without high-intensity physical activity, and its effect on CSF levels of the amyloidogenic peptide Aβ_42_. The main outcome was that patients with MCI subjected to a modulated diet, and who had a high-intensity physically active lifestyle, had higher levels of CSF Aβ_42_, than those without an active lifestyle. Furthermore, brain levels of Aβ_42_ have been shown in animal studies to be reduced in response to physical exercise (62). Aβ_42_ therefore appears to be a physiologically relevant biomarker that was not measured in any of the included studies likely due to difficulty of including CSF measures in study design, attributable to the discomfort of lumbar puncture.

### Normal aging

In previous studies on the effect of exercise in a population of healthy elderly individuals, a decrease in the metabolic biomarkers of cholesterol, HDL, and leptin (63) was described. Furthermore, the exercise group showed increased glucose sensitivity after intervention, compared to controls (63). Aging is connected with chronic low-grade inflammation, increased risk for disease, poor physical function and mortality (64). Exercise has been shown to decrease the levels of circulating inflammatory cytokines (65). The expected effects of exercise on biomarkers of metabolism and inflammation are similar between normal aging individuals and patients with dementia. In the study by Nascimento et al. (39, 41), where aged matched cognitive normal controls were studied, the effect of exercise on the inflammation biomarkers were not specific to either the dementia group or the control group. However, only the MCI group showed improved cognition. This could indicate a link between cognitive measures and alterations in the inflammation profile. One could speculate that the lack of effect on cognition seen in the control group could be due to the scale chosen for measuring cognition (MoCA). MoCA may not be sensitive enough to quantify cognition in a group that already performs well cognitively, as this group had high MoCA scores even before intervention, and thereby improvement in the controls group will not be detected.

BDNF has previously been investigated not only for its brain plasticity modulating effect in dementia patients, but also in subjects with depression (66) and in animal studies, high-intensity exercise has a modulating effect on BDNF (26). Studies have found that BDNF levels decline with age, and it has been shown to be associated with memory deficits (67). An up-regulation of BDNF would therefore be beneficial, and maybe act as a protecting factor against dementia and other memory deficiencies.

### Genetic risk factors

The effect of the known risk factor for AD, ApoE (68), was found not to have an effect for the outcome on biomarkers after an exercise intervention. Previous studies have indicated that that outcome of an exercise intervention could be ApoE genotype dependent (69). However, neither Eggermont et al. (37) nor Cheng et al. (35), found any significant difference in effect according to ApoE genotype.

A possibility to further explore the ApoE effect on AD could be to investigate the gene product of ApoE, the protein apoE. A recent study has indicated that low levels of apoE increases risk of AD (70). Currently there are no plasma markers for AD, and perhaps apoE may have the potential to be a ground-breaking new risk factor for AD.

### Exercise intervention

In regards to the exercise protocol, most studies applied an aerobic training, like walking, to investigate the effect on biochemical markers. The studies reviewed have applied varying training regimes, durations, and frequencies. This makes a direct comparison difficult. However, most of the studies applying a moderate-to-high-intensity aerobic exercise protocol, have found a significant effect on biomarkers, while low-intensity protocols did not show significant effects. This could indicate that the level of intensity of the aerobic exercise is important for achieving an effect of an exercise intervention.

The level of supervision for protocol adherence and intensity varied greatly among the reviewed studies. Overall supervision is necessary to ensure general adherence to the program and that exercise intensity is maintained, especially when it comes to moderate-to-high-intensity exercise, where the physical demands on the patients are far greater. For example, supervised training sessions with professional trainers and equipment, such as a pulse watch, can be used.

### Cognition

Another caveat worth considering is whether the effect of physical exercise on cognition is caused by measurable changes in biomarkers that reflect the pathophysiology of the disorder or whether exercise improves cognition through general improvement of brain function through other mechanisms. These could include up-regulation of neurotransmitters relevant for cognition, such as NE or increase in vascular endothelial growth factor (71). This remains to be determined.

Biomarkers are the main outcome assessed in this review article, but when studying dementias, cognitive measures have to be taken into account. It is unclear whether lack of cognitive assessment as a measured outcome is due to negative findings or that cognition went untested. Although a change in a biochemical markers with physical exercise does not imply a therapeutic effect on symptomatology, an objectively measurable effect on a relevant pathophysiological biochemical parameter will support the importance of an implementation of physical exercise as part of the treatment for dementia.

### Recommendations for sampling and analysis

Of all the studies that have been conducted on exercise in patients with dementia disease or cognitive impairment, only eight studies have included biomarkers as a part of their assessment. However, the effect on functional activity and quality of life are relevant to the patient and caregiver, useful biochemical measures of these effects are still lacking.

Our recommendation for investigating the effect of physical exercise effect on dementia at a biochemical level is to (1) investigate whether any metabolic pathways that can be altered by increased physical activity could also be involved in dementia, (2) measure molecules from these pathways that have a neuronal contribution, and distinguishing their neuronal-specific contribution from systemic contribution, (3) investigate proteins and pathways that are involved not only in the generation and maintenance of neurons, but also relevant for cognitive function in general, and (4) measure an broad cytokine effect on neuroinflammation, since exercise has a putative anti-inflammatory effect (72).

Due to the fact that many established diagnostic markers for dementia disease are measured in CSF, including Aβ_42_ (73), tau (74), p-tau (74), α-synuclein (75), and huntingtin (76), we would further recommend to include CSF assessment in future studies, as this better reflects cerebral biomarker levels.

In order to achieve valid biomarker measurements, the highest quality of samples for analysis are required, and we recommend that strict sampling processing and storage procedures are observed (77).

## Conclusion

Eight out of fifty-four exercise studies in dementia or MCI have investigated biochemical markers of the effect of exercise on dementia and MCI. There is an overall trend of beneficial effect of exercise on the selected biomarkers. However, there were no studies that investigated specific Aβ pathology, or pathological processes downstream of Aβ accumulation.

Future studies with greater samples size, more thorough exercise supervision, educated trainers and well-defined intensity measures, as well as various sampling protocols (blood, CSF, etc.) are required. Such studies are in progress (78) and will hopefully help to understand the beneficial effect of physical exercise on dementia.

## Author Contributions

Study conception and design: CJ, AS, and SH. Acquisition of data: CJ and SH. Analysis and interpretation of data: CJ, SH, GW, and AS. Drafting of manuscript: CJ, SH, GW, and AS. Critical revision: SH, GW, and AS.

## Conflict of Interest Statement

The authors declare that the research was conducted in the absence of any commercial or financial relationships that could be construed as a potential conflict of interest.

## Supplementary Material

The Supplementary Material for this article can be found online at http://journal.frontiersin.org/article/10.3389/fneur.2015.00187
