# Supplementary material for: Biochemical Markers of Physical Exercise on Mild Cognitive Impairment and Dementia: Systematic Review and Perspectives
Source: Front Neurol. 2015 Aug 26;6:187. doi: 10.3389/fneur.2015.00187 (PMC4549649; doi:10.3389/fneur.2015.00187)
Supplement: Supplementary file 1 [file datasheet_1.docx]

***Supplementary Material***

**Biochemical biomarkers in physical exercises effect on Mild Cognitive Impairment and dementia; systematic review and perspectives**

Camilla Steen Jensen^1*^, Steen Gregers Hasselbalch^1,2^, Gunhild Waldemar^1,2^, Anja Hviid Simonsen^1^

^1^Danish Dementia Research Centre, Department of Neurology, Copenhagen University Hospital Rigshospitalet, Denmark

^2^Department of Clinical Medicine, Copenhagen University, Denmark

*** Correspondence:** Camilla Steen Jensen, cand.scient. Biology, Danish Dementia Research Centre,

Department of Neurology, Copenhagen University Hospital, Rigshospitalet, Blegdamsvej 9. DK-2100 Copenhagen, Denmark

Camilla.steen.jensen@regionh.dk

**Supplementary Data**

**Supplementary Table 1.** Studies on the effect of physical exercise in MCI and dementia which did not include biomarkers as part of their study aim.

| Publication | Endpoint | Subjects | Intervention | Results |
| --- | --- | --- | --- | --- |
| (Aman and Thomas, 2009) | ADL, depression, agitation | 50 dementia^†^ | n=40, 15 min aerobic (walking) + 15 min resistance (1 lbs. weight) 3/ week * 3 weeks^€^ | ↑6-meter walk, ↓agitation |
| (Barnes et al., 2013) | Change in cognitive function (RAVLT, DSST, Trail A&B , verbal fluency, EFT, UFOV, modified MMSE) | 126 cognitive complaints^¥^ | Metal activity: DVD or computer, exercise activity: physical aerobic exercise (60-75% HRR) or stretching •. 60 min 3/week * 12 weeks. | Changes from baseline in all groups: ↑ DSST, ↑trail A, ↑UFOV, ↑ EFT |
| (Busse et al., 2013) | QOL, retention, cognition | 31 Huntington disease ^†^ | n=16, 1/week aerobic exercise• (55-75% HRR) 2/week exercise (walking)^€^ * 12 weeks | ↑MSC score |
| (Cadore et al., 2014) | GVT, TUG, verbal GVT and counting GVT, FICSIT-4, chair-raise, MMSE, BI | 18 dementia ^†^ | n=18, 8 weeks in total, daily walks/balance exercises + cognitive activities * 4 weeks and exercise as above + 2/week resistance (20-50% 1RM) training * 4 weeks^€^ | ↓fall risk, ↓TUG, ↑balance, ↑strength |
| (Canonici et al., 2012) | MMSE, NPI, depression, ADL | 32 AD^†^ | n=16, mixed aerobic exercise 60 min 3/ week * 26 weeks ^€^ | ↓NPI, ↑ADL, ↑balance |
| (Carvalho et al., 2015) | EEG, PD motor symptoms, functional capacity | 22 idiopathic PD^†^ | n=8, resistance training (80% 1RM) 30 min 2/week * 12 weeks. N=5, aerobic exercise (70 % HRR) 30 min 2/week*12 weeks. N=9, physiotherapy 30-40 min 2/week * 12 weeks | ↑EEG, ↑PD motor symptoms, ↑functional capacity |
| (Chang et al., 2011) | Balance, chair raise | 26 dementia^¥^ | n=26, stretching + walking 20-30 min 5/week *?^€^ | Trend: ↑ADL, ↑chair raise, ↑balance |
| (Coelho et al., 2013) | FSB, clock draw test, Symbol Search Subtest, gait, dual-task^£^ | 27 AD^†^ | n=14, aerobic + balance exercise 60 min 3/week * 16 weeks^€^ | ↑FSB, ↑Symbol search test. Controls: ↓dual-task, ↓clock draw test, trend ↓gait |
| (Cott et al., 2002) | ADL, 2 min test, London psychogeriatric ration test | 73 AD^†^ | n=30, walking + talking 30 min 5/week * 16 weeks^€^. n= 25 talking 30 min 5/week * 16 weeks^€^ | No significant results found |
| (de Andrade et al., 2013) | MCS, clock draw test, Symbol search test, FSB, dual-task, TUG, chair raise, balance | 30 AD^†^ | n=14, aerobic exercise (65-75 % HRR) + resistance + cognitive activity 60 min 3/week* 16 weeks^€^ | ↑clock draw, ↑balance, ↑dual task, ↑functional capacity |
| (Eggermont et al., 2010) | Sleep disturbances, night-time restlessness, daytime activity | 79 dementia^†^ | n=41, walking 30 min 5/week * 6 weeks, | No significant results found |
| (Garuffi et al., 2013) | ADL, lower limb strength, sock test | 23 dementia^†^ | n=17, resistance training 60 min 3/week* 16 weeks^€^ | ↑lower limb strength, ↑balance, ↑flexibility, ↑ADL |
| (Hagerman and Thomas, 2002) | 6-meter walk, speed over 6-meters, TUG | 23 dementia^†^ | n=23, resistance training (moderate) 2-3/week * 6 weeks^€^ | Non-significant ↑ gait time |
| (Hauer et al., 2012) | Leg strength (1RM), 5 chair raise, physical activity, max strength, physical function | 122 dementia^†^ | n=62, resistance training (70-80% of 1RM) 120 min 2/week * 12 weeks• | ↑ leg strength, ↑functional performance, ↑physical activity |
| (Hernandez and Stella, 2010) | MMSE, BBS, TUG, AGIBAL | 16 AD^†^ | n=9, aerobic exercise (60-80% HRR) + resistance training (moderate) 60 min 3/week*26 weeks• | Control group: ↓MMSE, ↓TUG, ↓BBS |
| (Kemoun et al., 2010) | ERFC, walking (speed, stride length, double limb support time) | 31 dementia (AD)^†^ | n=16, aerobic exercise (60-70% HRR) 60 min 3/week* 15 weeks^€^ | ↑ERFC, ↑walking speed, ↑stride length, ↓double limb support time |
| (Kovacs et al., 2013) | Fall risk, balance, TUG, POMA | 86 dementia^¥^ | n=32, walking 30 min 1/week + resistance training (light-to-moderate) 60 min 2/week * 52 weeks• | ↑balance, ↓ fall risk |
| (Kwak et al., 2008) | MMSE, ADL, cardiac output, strength, endurance, flexibility, balance, agility | 30 ♀ dementia^¥^ | n=15, aerobic exercise (30-60% HRR) + resistance training 30-60 min 2-3/week* 12 weeks^€^ | ↑MMSE, ↑ADL, ↑cardiac output, ↑muscle strength, ↑muscle endurance, ↑flexibility, ↑agility |
| (La Rue et al., 2015) | MMSE, mood, physical fitness, QOL | 29 dementia^¥^ | n=29, exercise? 90 min 1/week* ? ^€^ | ↑Physical fitness →MMSE, ADL, mood ↓QOL |
| (Littbrand et al., 2009) | ADL | 191 (100 dementia^¥^) | n=91 (both ±dementia) resistance training 2.4/week * 12 weeks^€^• | ↑ADL |
| (McCurry et al., 2012) | Total wake time, sleep percent, number of awakenings, SDI | 132 AD^†^ | N=32, walking 30 min/day, n=34, light therapy 60 min/day, n=33 walking 30 min/day+ light therapy 60 min/day * 8 weeks• | Walking+ light therapy ↑total wake time |
| (Nagamatsu et al., 2013) | STROOP, Trail, verbal digit test, fMRI, 6-min walk test, general mobility + balance | 86 ♀ MCI^†^ | n = 28, resistance training 60 min 2/week* 26 weeks, n= 30 Aerobic exercise (70-80% HRR) 60 min 2/week*26 weeks, n= 28 balance training 60 min 2/week*26 weeks | Resistance training ↑STROOP ↑fMRI. Aerobic exercise: ↑balance ↑mobility ↑Cardiac output |
| (Nascimento et al., 2012) | Neuropsychiatric test, Pfeffer function activities | 20 AD^†^ | n=10 aerobic exercise (60-80%HRR)60 min 3/week*26 weeks• | Controls group: ↓in neuropsychiatric, ↓ functional |
| (Roach et al., 2011) | Mobility, 6-meter walk | 82 AD^†^ | n=28, walking+ balance training 15-30 min 5/week*16 weeks^€^. n=29, walking 15-30 min 5/week*16 weeks^€^. | Walking + balance training ↑chair raise, |
| (Rolland et al., 2007) | ADL, 6-meter walk, TUG, balance, behavior disturbances, depression, nutritional status | 134 AD^†^ | n=67, walking + balance 60 min 2/week*52 weeks^€^ | Walking+ balance slower ↓ADL, ↑5-meter walk speed |
| (Santana-Sosa et al., 2008) | ADL, muscle strength, flexibility, balance, endurance | 16 AD^†^ | n=8, walking + stretching 75 min 3/week*12 weeks• | ↑muscle strength, ↑ flexibility, ↑agility, ↑balance, ↑endurance, ↑gait, ↑ADL |
| (Schwenk et al., 2014) | gait | 61 dementia^†^ | N=20, resistance training (70-80% 1RM)120 min 2/week*12 weeks• | ↑speed, ↑cadence, ↑stride length, ↑stride time |
| (Schwenk et al., 2010) | Gait, cognitive performance, dual task (either forward or backwards calculation) | 61 dementia^†^ | N=20, resistance training (70-80% 1RM)120 min 2/week*12 weeks• | ↑dual task backwards calculation |
| (Smith et al., 2013) | fMRI, Neuropsychological test battery, list-learn task | 17 MCI^†^ + 18 cognitive normal^†^ | N=35, aerobic exercise (50-60% HRR) 30 min 4/week*12 weeks^€^ | MCI: ↑list-learn task. Both: ↑cognitive function ↑fMRI |
| (Sobel, 2015) | Short-term memory concentration, BNT, word-list recognition task | 50 AD^†^ | N=50, walking 20 min once^†^. 4-7 days wait, n=50, bingo 20 in once^†^ | Bingo: ↑BNT, ↑Word list recognition |
| (Stevens and Killeen, 2014) | REPDS, clock draw | 75 dementia^†^ | N=24, aerobic exercise (light) 30 min 3/week*12 weeks^€^. N=21 social meeting controls 30 min 3/week*12 weeks^€^ | Slower ↓clock draw, ↑REPDS |
| (Tappen et al., 2007) | 6-min walk | 65 AD^†^ | n= 26, walking 30 min 3/week*16 weeks^€^. n= 21 walking + talking 30 min 3/week*16 weeks^€^. n= 24 talking 30 min 3/week*16 weeks^€^ | Walking Slower ↓functional mobility |
| (Teri et al., 2015) | Physical health, physical function, depression | 153 dementia^¥^ | N=68, aerobic exercise (+ resistance training) + behavior activity 30 min/day*12 weeks^€^. Exercise 30 min/day*12 weeks^€^ | Exercise + behavior activity: ↑restricted activity, ↑ physical function, ↓depression |
| (Thomas and Hageman, 2003) | Strength, chair raise, gait, TUG | 28 dementia ^†^ | N=28, resistance training (light-moderate) 3/week*6 weeks^€^ | ↑strength (hand grip, quadriceps), ↑TUG, ↑chair raise, ↑ gait performance |
| (Toulotte, 2003) | TUG, CSR, balance, gait speed | 20 dementia^†^ | N=10, resistance training (moderate) 45 min 2/week*16 weeks^€^ | ↑balance, ↑gait speed, ↑flexibility, ↑mobility |
| (Uemura et al., 2013) | TUG, MMSE, Trail, depression | 44 MCI^†^ | N=44, aerobic exercise (40-60% HRR) + resistance training 90 min 2/week*26 weeks | ↑general cognition, ↑general fitness |
| (Varela et al., 2012) | MMSE, TUG | 47 MCI^†^ | N=17, aerobic exercise (40% HRR) 30 min 3/week*12 weeks•. n=15, aerobic exercise (60% HRR) 30 min 3/week*12 weeks• | Trend ↑TUG, trend ↑MMSE |
| (Venturelli et al., 2011) | ADL, 6-minut walk test, MMSE | 21 AD^†^ | N=11, walking 30 min 4/week*26 weeks^€^ | Slower ↓MMSE, ↑6-minut walking test, ↑ADL |
| (Vital et al., 2012) | MMSE, BCB, clock draw, Verbal fluency test | 34 AD^†^ | N=17, resistance training (moderate-to-high) 60 min 3/week*16 weeks^€^ | No significant results found |
| Wang et al., 2010 | Sleep quality, general health, depression | 34 CVD^†^ | N=17, aerobic exercise (Tai Chi) 50 min 1/week*12 weeks^€^ | ↑sleep quality, ↓depression, ↓insomnia, ↓general health |
| Wei and Ji, 2014 | MMSE, ADL | 60 MCI^†^ | N=30, aerobic exercise + resistance training (moderate) 30 min 5/week*26 weeks^€^ | ↑MMSE, ↑ADL |
| Yágüez et al., 2011 | CANTAB | 27 AD^†^ | N=15, resistance training (light/stretching) 120 min 1/week*6 weeks^€^ | ↑sustained attention, ↑visual memory, ↑working memory |
| Yerokhin et al., 2012 | EEG, ERP, STROOP, FOME, call-recall test | 22 (13 dementia^†^ + 9 non-demented) | N=22, resistance training (light) 45 min 3-5/week*10 weeks^€^ | ↑Verbal memory |
| Yu et al., 2011b | Cardio fitness, VO2 peak, Blood pressure, ECG, HRR, peak heart rate | 4 ♂ AD^†^ | N=4, aerobic exercise (40-70% HRR) 10-30 min/week *8 weeks^€^ | Men with AD can engage un aerobic exercise |
| Yu et al., 2011a | Cardio fitness, lower limb function | 8 AD^†^ | N=8, aerobic exercise (moderate) 45 min 3/week*26 weeks^€^ | ↓heart rate, ↑improved cardio fitness |
| Yu and Swartwood, 2012 | Adherence | 10 AD^†^ | N=10, aerobic exercise (65-75% HRR) 45 min 3/week*26 weeks^€^ | Aerobic exercise feasible for patients with AD |

ADL: Activity of daily living, HRR: heart rate reserve, RAVLT: Rey Auditory Verbal Learning Test, DSST: Digit Symbol Substitution Test, Trail A: trail-making test part A, Trail B: Trail-making test part B, EFT: Eriksen Flanker test, UFOV: Useful Field of view, MMSE: Mini-mental state examination, QOL: Quality of life, MSC: Mental Component Summary, GVT: 5-m habitual gait test, TUG: Time-up-and-go, FICSIT-4:FICSIT-4 test of static balance (parallel, semi-tandem, tandem and one-legged stance tests), BI: Barthle Index, 1RM: One repetition maximum, NPI: Neuropsychiatric Inventory caregiver Distress Scale, AD: Alzheimer’s disease, EEG: Electroencephalogram, PD: Parkinson’s disease, FSB: Frontal Assessment Battery, MSC: Montreal Cognitive assessment, BBS: Berg Balance scale, AGIBAL: agility/dynamic balance item of the American alliance for health, physical education, recreation and dance (AAHPERD), ERFC: rapid evaluation of cognitive function, the French version, POMA: the performance oriented mobility assessment scale, SDI: rating of sleep, fMRI: functional magnetic resonance imaging, CSR: chair sit-and-reach, TMT: BCB: Brief cognitive battery, CVD: Cerebral Vascular Disorder, CANTAB: the Cambridge Neuropsychological Test Automated Battery, ERP: Event related potential, FOME: full object memory evaluation, ECG: electrocardigraphy,VO2 peak: peak oxygen uptake, BNT: Boston Naming Test, REPDS: revised elderly person disabilities scale.^†^: Diagnosed according to international guidelines, ^¥^: non-diagnosed or not mentioned, ^€^: Supervised by caregiver or nursing facility staff or not mentioned, •: supervised by trainer or physiotherapist, ^£^: gait + counting.

## Supplementary Table 2. Data extraction form

| Publication | Endpoints | Subjects | Intervention | Results |
| --- | --- | --- | --- | --- |
| First author and year | Endpoints measured, e.g. cognition, gait etc. | Number of subjects used in study and diagnoses | Number of subjects participating in intervention of physical exercise, and type and duration of intervention e.g. duration, intensity/load, supervise/non-supervised | Results found for the exercise groups compared to control, were ↑means an increase or an improvement, ↓means a decrease, or worsening of condition, →means stabilized |
